# Cerebral Syphilis

**Published:** 1893-04

**Authors:** Mathew M. Smith

**Affiliations:** Resident Physician City Hospital, Austin, Texas


### DANIEL'S TEXAS MEDICAL JOURNAL.

ESTABLISHED JULY, 1885.

PUBLISHED MONTHLY.— SUBSCRIPTION $2.00 A YEAR.

Vol. VIII. AUSTIN, APRIL, 1893. No. 10.

### Original Contributions.

For Daniel's Texas Medical Journal.

### CEREBRAL SYPHILIS:

BY MATHEW M. SMITH, B. S., M. A., M. D., RESIDENT PHYSICIAN CITY HOSPITAL, AUSTIN, TEXAS.

[Read at Austin Distrist Medical Association, March, 1893.]

CEREBRAL SYPHILIS like many other diseases of the nervous system is regular in its appearance, difficult often of diagnosis and varied in its symptomatology. Syphilis as you know, affects man's whole organism, and is therefore constitutional. The infection enters the system by means of the blood vessels and lymphatics, attacking primarily the connective tissue, as a low grade inflammation with cell growth, forming granulation tissue and later involving the deeper and more important structures of the body; and as a result of these changes the nervous system may become involved; by means of deposits into the meninges, substances of the brain and cord and the peripheral nerves.

The principal anatomical lesions of brain syphilis that occur, are gummata, obliterating endarteritis and a pachymeningitis.

*The intra-cranial gummata*, usually originate in the dura or pia mater, and extend; but they rarely begin in the substance of the brain.

At first there is formed simply, soft, granulation tissue, but later central caseation and fibroid metamorphosis take place.

These gummata may grow into the brain substance, and if at the base of the brain they develop and fill in all the interstices around the chaism, the crura and pons, and when they extend into the adjacent brain tissue we have softening as a result.

*The obliterating endarteritis*, is not uncommon, and usually occurs at the base of the brain The affected artery becomes hardened and thickened, by a new growth of fibrous tissue, beginning in the intima and increasing until the lumen is diminished to such an extent, that only one half or one fourth of the required amount of blood passes through it, or the artery may be entirely obliterated by this new growth, and that portion of the brain substance supplied by it degenerates.

Or thirdly, *the Meninges* may be the seat of inflammatory action; and a meningitis or pachymeningitis be the result, and there is formed, rather tough and thickened patches, or the inflammation may extend into the brain substance and thereby cause softening. As to the cause of Cerebral Syphilis, we all know the direct cause is syphilis, either inherited or acquired, and most usually the latter; and the attack may occur within a few months after the infection or it may be years later. But why it is, that one portion of the nervous system is selected as the seat of attack, in preference to another we do not know. But it is plausible to believe, it lays hold of that part rendered weakest by some lesion or lowered state of vitality; anything that has a tendency to reduce the general health will act indirectly as an exciting cause for the outbreak; such as over-brain work, venereal and alcoholic excesses, exposure, etc.

SYMPTOMS.—The onset may be very sudden, as an apoplectic attack, an epileptic convulsion, a paralytic stroke or a maniacal seizure. But usually it comes on gradually with prodromes, such as severe nocturnal headaches, paroxysmal in character, vertigo, confusion of mind, irritability of temper, melancholia and later, fainting spells, weakness of limbs, difficulty in speech, unequal pupils, strabismus epileptic or apoplectic attacks and even maniacal appearances. The symptoms in each individual case will necessarily vary, with the seat and the extent of the lesion. If basal deposits, there is usually inco-ordination, excessive vertigo, nausea, swoolen eyelids, epistaxis, etc. If pressure is made by gummata in this locality, affections of the cranial nerves, facial paralyses, neuralgias, disorders of vision, etc. And involvement of the motor area will produce mortor spasm, weakness, hemiplegia, and frequently epileptic convulsions. If occlusion of cerebral arteries occurs, the symptoms resemble thrombic softening, and the brain symptoms due to pressure, are similar to those seen in usual tumors of the brain.

I shall enumerate more in detail, the peculiar somnolence, paralyses, epilepsy and coma that are largely pathognomonic of cerebral syphilis.

SOMNOLENCE.—A peculiar condition which is very characteristic when present. The patient is often able to work; but may fall asleep at his task, and become very sleepy in the evenings. In the more advanced stages the mental symptoms are peculiar. Patient may be aroused but thinks slowly and speaks with difficulty; may get out of bed and wander without a knowledge of his whereabouts, and cannot find his bed on returning. May pass his secretions in one corner of the room or elsewhere, not for want of control, but because he feels to be in proper place. He is truly a restless, nocturnal rambler. This somnolent condition may last for weeks. In its excessive development, there appears evidences of brain softening.

PARALYSES.—Their mode of occurrance shows great variation. They may appear gradually or suddenly, with or without an apoplectic or epileptic attack. Special and tansient palsies may occur, present to day and absent to-morrow; numbness and formication. The special senses may become involved, aphasia often is present in the more advanced stages of the disease.

EPILEPSY.—Of frequent occurence in cerebral syphilis, appearing in the two forms; grand and petit mal, headache usually precedes an attack. The symptoms of the severe form are similar to those occurring in non-specific epilepsy. A sudden loss of consciousness, tonic, followed by clonic spasms, foaming at the mouth, stertorous respiration; in some cases consciousness soon returns, while others remain unconscious and stupid for hours and even for days; a mild form may occur; there is a twitching of one side of the face, tongue drawn to one side, giddiness, trembling, weakness or cramps. Yet there may be only a rigidity, even no convulsion at all, patient while talking becomes unconscious and stares vacantly; this soon passing away and he proceeds with his usual avocation.

COMA—May occur with premonitory symptoms, but often comes on with sleep and is noticed the following morning. The patient is pale, stupid, pupils contracted and irregular, divergent strabismus, reflex sensibility of conjunctiva abolished, marked relaxation of muscles, more or less anæsthesia and diminished reflexes, incontinence of urine and fæces, pulse ranging from 40 to 60, respiration slow and shallow. The initial lasts from two to five days, usually by the tenth day the patient has recovered or died. In the final stage the symptoms increase in severity until death. In recovery the attacks may return again. Syphilitic coma is comparatively of rare occurrence.

DIAGNOSIS.—We now come to an element about which there is great variability. One case may present typical symptoms, with a well-known syphilitic history; while another rests in obscurity, with a variety of indefinite symptoms. We must inquire into the history of all cases. Ocular palsies, epileptic convulsions after thirty years, headaches, partial failure of memory, numbness and weakness, hemiplegia, with exaggerated reflexes, etc., all go to make a clear case of brain syphilis. An excessive exaggeration of the deep tendon reflexes, of both upper and lower extremities, between the ages of eighteen and forty, is almost pathognomonic, when with a doubtful history. We must bear in mind those diseases which resemble the one in question, and isolate in that way. Where there is any doubt, give your patient the benefit of the doubt and give scruple doses of potassium iodide three times a day, and if no iodism is produced, as a rule, your diagnosis is correct.

PROGNOSIS—Is not unfavorable if treatment is properly made use of at the onset. Yet there are many cases where treatment does not do any good, and relapses may occur.

TREATMENT.—The mixed treatment is undoubtedly the best; either give together or alternate. Use the iodide in large and increasing doses, and the mercury to tolerance. The following rules should obtain in all cases of cerebral syphilis: 1st, Use all possible expediency; 2d, Employ as energetic means as are feasible; 3d, Continue treatment during the entire attack and for a long time after the disappearance of all symptoms.

CASE.—A. J., negro woman, age forty years, mother of six children; three living, one died at the age two years, with supposed measles, the other two were miscarriages. She gives some history of an old case of syphilis; scars over various parts of the body; about four years ago she had a violent eruption upon the face and neck, which later disappeared and left numerous scars over that region. A year or two after the disappearance of the eruption while washing, she suddenly lost consciousness and fell, stricking her head against the stove, making a scalp wound over the left parietal bone. After a day and night she regained consciousness and returned to her usual health, which continued until six months later, when she had a second attack, very much like the first, but upon recovery she noticed some weakness and loss of power in the right hand. A few months later a third attack followed and with it an increase of the loss of power in the right hand and arm, and finally she had a fourth attack and remained unconscious for three or four days, and upon recovery she found almost complete loss of power in the right arm and leg. She has had these attacks ever since, occurring with no regularity. They are epileptiform in character. Just preceding their onset she experiences a trembling and drawing sensation in the hand, and it spreads over the right side of the body, and a typical convulsion is the result. It disappears after an hour or two and leaves her in that stupid condition common in epilepsy, and after a sleep she returns to her normal health, except some nausea and weakness which disappear in a day or two. Examination shows the deep tendon reflexes increased and upon the right side much exaggerated, with decided right ankle clonus. She has a scar over the left parietal bone, which shows tenderness upon pressure, but I think no depression. She suffers from constipation. Some opacity upon the right cornea, but no ophthalmic examination was made. Usually little headache, an irritable stomach often, sleeps soundly at night, appetite usually good. About two years ago she was a helpless hemiplegic, but under treatment she has regained power until she can walk unaided about the room, and the epileptic attacks are much milder and are not so frequent. The best results have been obtained by the mixed treatment, with other remedies to meet any symptom that might arise.

In this case, with the syphilitic history, two miscarriages, corneal opacities, the epileptic convulsions with hemiplegia, and a decided benefit with treatment, make an unmistakable case of cerebral syphilis. I believe from the symptoms of this case that a syphilitic gumma had formed in the dura or pia mater, over the cortical surface of the brain, and by growing made pressure over the arm and leg centers, thereby causing the epileptic attacks and hemiplegia. These gummata being more or less fibrous in their nature, may not be completely removed by appropriate treatment. .In the earlier stages of this case, no treatment was had, when the best results could have been obtained, and now, I think, there must be a degeneration of a certain amount of brain substance which indicates a permanent lesion.

